# Decellularization Techniques for Tissue Engineering: Towards Replicating Native Extracellular Matrix Architecture in Liver Regeneration

**DOI:** 10.3390/jfb14100518

**Published:** 2023-10-16

**Authors:** Ishita Allu, Ajay Kumar Sahi, Meghana Koppadi, Shravanya Gundu, Alina Sionkowska

**Affiliations:** 1 Department of Biomedical Engineering, University College of Engineering (UCE), Osmania University, Hyderabad 500007, India; saiishitaallu@gmail.com (I.A.); kmeghana203@gmail.com (M.K.); 2 School of Medicine, McGowan Institute for Regenerative Medicine, University of Pittsburgh, Pittsburgh, PA 15219, USA; ajaysahi15@gmail.com; 3 Faculty of Chemistry, Nicolaus Copernicus University in Torun, Jurija Gagarina 11, 87-100 Torun, Poland; 4 Faculty of Health Sciences, Calisia University, Nowy Świat 4, 62-800 Kalisz, Poland

**Keywords:** decellularization, liver, tissue engineering, extracellular matrix, scaffold

## Abstract

The process of tissue regeneration requires the utilization of a scaffold, which serves as a structural framework facilitating cellular adhesion, proliferation, and migration within a physical environment. The primary aim of scaffolds in tissue engineering is to mimic the structural and functional properties of the extracellular matrix (ECM) in the target tissue. The construction of scaffolds that accurately mimic the architecture of the extracellular matrix (ECM) is a challenging task, primarily due to the intricate structural nature and complex composition of the ECM. The technique of decellularization has gained significant attention in the field of tissue regeneration because of its ability to produce natural scaffolds by removing cellular and genetic components from the extracellular matrix (ECM) while preserving its structural integrity. The present study aims to investigate the various decellularization techniques employed for the purpose of isolating the extracellular matrix (ECM) from its native tissue. Additionally, a comprehensive comparison of these methods will be presented, highlighting their respective advantages and disadvantages. The primary objective of this study is to gain a comprehensive understanding of the anatomical and functional features of the native liver, as well as the prevalence and impact of liver diseases. Additionally, this study aims to identify the limitations and difficulties associated with existing therapeutic methods for liver diseases. Furthermore, the study explores the potential of tissue engineering techniques in addressing these challenges and enhancing liver performance. By investigating these aspects, this research field aims to contribute to the advancement of liver disease treatment and management.

## 1. Introduction

The field of tissue engineering has led to a considerable breakthrough in hepatic decellularization. This has made it possible to generate functioning liver tissues, which may have implications in transplantation as well as tissue regeneration. Decellularization corresponds to the precise removal of donor liver cells, resulting in an acellular scaffold that preserves the complicated three-dimensional architecture and extracellular matrix composition of the organ [1,2,3]. This scaffold can then be repopulated with patient-specific hepatocytes or hepatic stem cells, which could facilitate the construction of bioengineered hepatic structures [4,5]. This novel strategy has the potential to address the organ donor shortage and advance the understanding of issues pertaining to the liver, such as the causes of various disorders and the corresponding therapeutic solutions [4,6,7].

## 2. Anatomy of the Liver

The liver is the largest internal organ in the human body, accounting for about 2% of the total body weight of an average adult. It is a wedge-shaped organ located just beneath the diaphragm in the right hypochondrium, extending into the epigastric region to reach the left hypochondriac region in the abdominal cavity [8]. Hepatic tissue consists of two cell types: parenchymal cells called hepatocytes and cholangiocytes (biliary epithelial cells) and non-parenchymal cells encompassing Pit cells, Kupffer cells, hepatic stellate/fat-storing cells, and sinusoidal endothelial cells [9]. One of the primary roles of hepatocytes is the secretion of proteins into the blood (albumin and clotting factors). Cholangiocytes line the biliary tree’s ducts and interact with the hepatocytes via the canals of Hering. Cholangiocytes create 30% of total bile flow and significantly interact with bicarbonate and other molecules. Hepatic stellate cells are a storehouse for Vitamin A. Kupffer cells are specialized macrophages capable of phagocytizing foreign materials, generating pro-inflammatory cytokines, and presenting antigens. Located beneath the endothelial cells and fibroblasts are the Pit cells, which account for a small proportion of the non-parenchymal cell population and are natural killer cells [10,11]. The parenchymal cell population is estimated to be around 80% of the hepatic tissue volume, while non-parenchymal cells account for approximately 6.3% (2.8% endothelial cells, 2.1% Kupffer cells, and 1.4% stellate cells) [12]. 

Being a highly vascular organ, the liver receives about 25% of the total cardiac output and has a unique angioarchitecture with a dual blood supply from two afferent vessels. The hepatic artery, arising from the celiac trunk, is responsible for 25–30% of the liver’s blood supply, which is rich in oxygen. Contributing to the remaining 70–75% of blood flow is the portal vein, formed by the junction of the splenic vein and the superior mesenteric vein. Portal blood is enriched with monosaccharides and amino acids absorbed by the intestine from the splanchnic circulation, but it also contains bile salts, bilirubin, and GI hormones [8,13]. Toxins and metabolic waste are brought into the liver by the portal blood for detoxification before this blood enters the systemic circulation.

**Figure 1:**
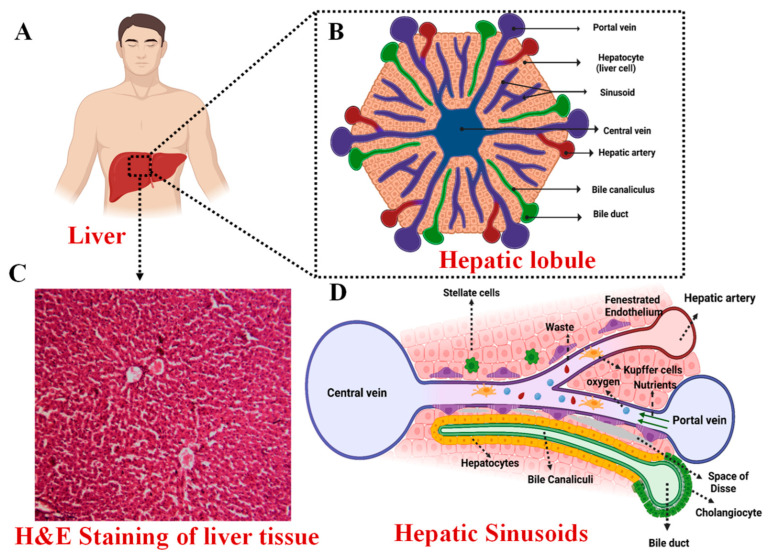
Liver architecture: (**A**) Illustration of the Macroscopic Liver Structure (**B**) Detailed Description of Liver’s Lobular Architecture, encompassing vascular and biliary elements (**C**) Microscopic histological examination of liver tissue staining (**D**) composition and architecture of the Liver Lobule structure [10].

The liver is divided, rather unequally, into two parts called lobes: a larger right lobe and a smaller left lobe. Topographically, the falciform ligament demarcates the right lobe from the left, but from a functional standpoint, this division is inaccurate. The medial part of the left lobe is anatomically placed to the right of the falciform ligament, centered on the anterior branches of the left portal vein [8,9]. Each hepatic lobe consists of numerous lobules, which are the structural and functional units of the liver tissue. Figure 1 shows the gross structure of the liver and its lobular architecture. Each lobule is constructed around a central vein with rows of hepatocytes arranged in cellular plates radiating outwards, forming a roughly hexagonal pattern. The lobules are surrounded by multiple portal triads, which consist of a branch of the portal vein, a branch of the hepatic artery, and a tributary of the bile duct [14]. Hepatic plates are typically two cells thick and are separated by small bile canaliculi, which empty into bile ducts in the fibrous septa that separate adjacent liver lobules. The fibrous septa also contain small portal venules that receive blood from the portal vein. Hepatic cells are constantly in contact with portal venous blood. Interlobular septa contain hepatic arterioles, which feed arterial blood to the tissues between the lobules. Adjacent plates are divided by sinusoids, which are blood passages lined with endothelial cells and contain the Kupffer cells [15]. The branches of the hepatic artery and portal vein open into the sinusoids, while the sinusoids open into the central vein. Ultimately, the central vein drains into the vena cava via the hepatic vein. Large pores in the endothelium lining allow plasma substances to reach the space of Disse, which interact with lymphatic vessels in the septa. The lymphatics drain excess fluid from these areas, and plasma proteins can also diffuse into them [16,17]. 

A remarkable feature of the hepatic tissue is its regenerative capability in that the organ can be restored completely even after significant tissue loss from partial hepatectomy, where two-thirds of the liver tissue is lost or after an acute liver injury as long as it is not aggravated by inflammation or viral infection [16]. After excision, the remaining liver expands in mass upon the replication of hepatocytes, facilitated by the hepatocyte growth factor (HGF), to compensate for the lost tissue. Once the original volume and size of the liver are restored, the hepatocytes cease to replicate and revert to their usual dormant state [18]. 

## 3. Functions of the Liver

The liver’s functions encompass a wide range of metabolic, detoxification, regulatory, and synthesis processes, which are crucial for maintaining the body’s homeostasis [19].

### 3.1. Metabolic Functions

Through glycogenesis, the liver regulates glucose in the body by converting and storing it in the form of glycogen. Certain aspects of lipid metabolism occur mainly in the liver, such as the synthesis of cholesterol, essential lipoproteins, and triglycerides and the oxidation of fatty acids via β-oxidation, generating energy for cellular processes. The liver also governs protein metabolism by synthesizing albumin, clotting factors, and globulins. It also participates in the deamination of amino acids and removes toxic ammonia through the urea cycle [20]. 

### 3.2. Bile Production

Bile is a fluid composed mainly of water, bile salts, electrolytes, phospholipids, bilirubin, and bile salts, among other substances. Produced by the hepatocytes, it helps excrete material that the kidneys do not eliminate and aids in the absorption and digestion of lipids. Bile is secreted into the bile canaliculi, where it travels to the larger ducts to end up in the duodenum or stored in the gallbladder. In the duodenum, it undergoes enterohepatic circulation, where it performs its job in the bowel, and the bile components are expelled in the feces [21]. The by-products after detoxification of drugs such as penicillin, sulfonamides, erythromycin, and more are excreted from the body through bile [13,22].

### 3.3. Bilirubin Metabolism

Hemolysis represents the intricate process wherein red blood cells (RBCs), having fulfilled their remarkable lifespan of 120 days, undergo breakdown. This degradation occurs at multiple locations, including the spleen, bone marrow, and liver [13]. Heme is broken down into biliverdin, which is reduced to unconjugated bilirubin. The unconjugated bilirubin is carried through albumin into the liver via the circulatory system. To become hydrophilic, unconjugated bilirubin is conjugated via the uridine diphosphate glucuronyltransferase (UGT) system in a phase II process. The newly conjugated bilirubin is subsequently released into the bile canaliculi or dissolves in the circulation, where it is filtered for elimination by the kidneys [23,24].

### 3.4. Other Functions

The liver regulates the synthesis of clotting factors and almost all the plasma proteins of the body, such as albumin, protein C, and protein S, to name a few. Additionally, the liver performs modification and excretion of hormones such as estrogenic, thyroxine, cortisol, and aldosterone. The liver is also an important site of the body’s immune system and immune-mediated damage induced by malignant, infectious, and autoimmune stimuli [16,22].

## 4. Prevalence of Liver Diseases

Accounting for over 4% of deaths worldwide, liver diseases are the eleventh leading cause of mortality and the fifteenth leading cause of disability-adjusted life-years (DALYs) [25]. Notably, out of the two million deaths attributed to liver-to-liver dysfunction, two-thirds afflict men. Liver disease has the greatest impact on the young, as it stands 12th top cause of DALY’s among individuals aged 25 to 49 years [26,27]. Cirrhosis is the most prevalent chronic liver disease, characterized by the hepatic tissue being replaced by dense scar tissue. This condition arises from recurring liver injury, necrosis, and inflammation, with culprits including hepatitis B and C, alcoholism, and non-alcoholic fatty liver disease (NAFLD) [11]. The consumption of alcohol elevates the risk of liver disease-related mortality by a staggering 260-fold, cardiovascular mortality by 3.2-fold, and cancer mortality by 5.1-fold. Notably, the number of deaths linked to NAFLD has witnessed a twofold increase over the past three decades. In 2020, viral Hepatitis B and Hepatitis C caused about 1.1 million deaths. The global prevalence of acute and chronic liver diseases is expected to increase significantly in the future [28].

To date, the ultimate remedy for addressing chronic liver diseases, particularly end-stage liver disease, is orthotopic transplantation. In 2020, a total of 129,681 solid organs were successfully transplanted across the globe, with liver transplants accounting for 25.1% of these cases [27]. However, there is still a huge discrepancy between the number of patients awaiting liver transplantation and that of donors, and a substantial number of people die while still on the waiting list. Furthermore, patients undergoing liver transplantation are highly susceptible to post-surgical complications such as infections, while the long-term effects include cardiovascular events, malignancies, and metabolic complications associated with immunosuppressive therapy. Strategic pre-transplant work-up and post-operative management are required in order to minimize the incidence of such complications, but it is a highly challenging task [26,27,29]. These limitations associated with organ transplantation necessitated the need for alternative therapeutic approaches such as cell therapy, bioartificial liver devices, organ-on-chip technology, and the bioengineering of hepatic tissue in vitro using primary or stem cells seeded into three-dimensional scaffolds [30].

Over the past few years, significant progress has been made toward the utilization of tissue engineering and regenerative medicine principles to restore liver functionality. Liver tissue engineering aims to produce functional liver tissue seeded with hepatic cells, followed by successful implantation into a patient with chronic liver disease [31]. Key variables that must be optimized for a successful hepatic tissue graft include selecting the optimal cellular sources, using the appropriate biomaterials for the scaffold, and locating suitable implantation sites [32]. For fabricating scaffolds mimicking vasculature and complex microarchitecture of the liver, bottom-up approaches such as 3D bioprinting, bio-microelectromechanical systems (“bioMEMS”), and 3D molding have shown promising results. However, it is challenging to accurately engineer macro-scale liver tissues using bottom-up approaches because of their inability to recreate the liver-specific extracellular matrix, its components, the controlled distribution, interconnectivity, and geometry of the pores [10,30].

## 5. Decellularized Extracellular Matrix Scaffolds

Decellularized ECM scaffolds have attracted the attention of researchers because of their biocompatibility properties, reduced risk of rejection due to the immune response, and similar mechanical and chemical properties to that of the native tissue or organ [33]. Such scaffolds are prepared using the decellularization technique, which involves the removal of the cellular or nuclear components while preserving the structural and functional proteins of the ECM. Cellular components and antigens are removed to prevent recognition by the immune system and cause an inflammatory response [34]. The proteins are preserved as they aid in cell adhesion, proliferation, and differentiation and also provide a natural environment for the stimulation of cell growth and, therefore, serve as an ideal material in tissue engineering [35,36,37,38].

The first successful account of liver decellularization was reported in 2004 when the researchers specifically explored a decellularized, porcine, liver-derived biomatrix as a bioresorbable scaffold for primary hepatocytes [39]. The research field has since demonstrated that decellularization is a relatively superior method for acquiring natural scaffolds compared to previous techniques, as it effectively preserves the native extracellular matrix (ECM) of the decellularized tissue [32,40]. Liver decellularization aims to preserve the major ECM components, including laminin, fibronectin, collagen type I, III, and IV, and proteoglycans.

### Hepatic ECM

**Figure 2:**
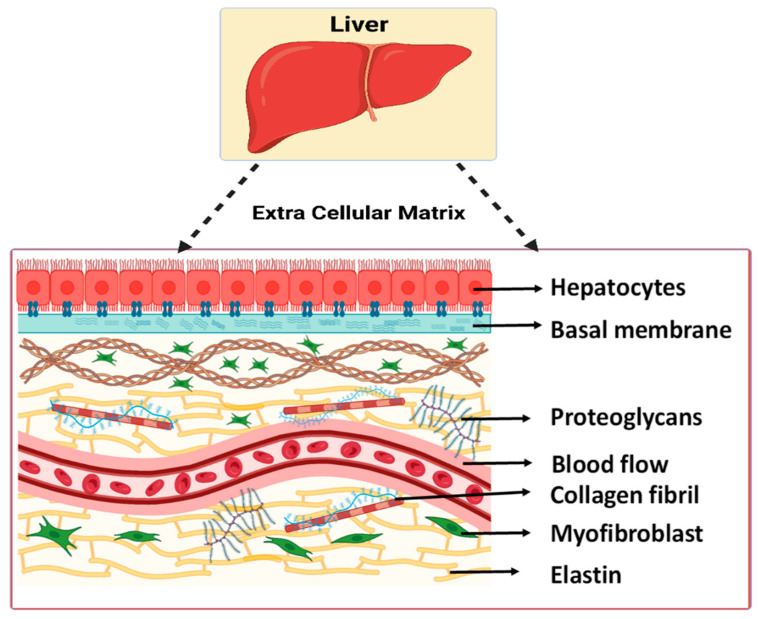
Schematic illustration of the major components of the hepatic ECM [50].

Most organs comprise comparable ECM components, although the concentrations and ratios differ [41]. The ECM is composed of two structures that are morphologically and biochemically different. They include the basement membrane and the interstitial matrix [42]. The basement membrane forms thin acellular layers that connect the epithelium to the interstitial matrix. The liver is characterized by a less dense basement membrane facilitating easier diffusion between plasma and the organ [41,43]. It is primarily composed of laminin, heparan sulfate proteoglycan (Perlecan), and collagen IV [5,44,45]. However, it is found to be absent in the parenchyma and sinusoids of healthy livers [5,44,46,47,48]. The interstitial matrix of the liver is primarily composed of collagen and fibronectin. The principal collagen types identified are Type I and Type III [5,47,49]. Figure 2 indicates the principal components of the liver ECM.

The liver ECM, accounting for 16% of the volume of the tissue, plays a vital role in cell-matrix adhesion, creating the milieu essential for hepatocyte growth, regulating tissue development, and the polarization of cells. ECM proteins trigger intracellular signaling by interacting with cell adhesion molecules such as integrins. These molecules play an important role in regulating cell differentiation, proliferation, migration, and gene expression [51,52]. Hence, it is crucial that the engineered scaffolds effectively mimic the innate microenvironment that supports the aforementioned phenomenon without eliciting immune-mediated rejection.

Decellularization can be achieved by various physical, chemical, or enzymatic methods, and the resultant ECM scaffold is sterilized before implantation [53]. The sterilization technique depends on the application considered, and it is performed either using irradiation or exposure to certain chemical agents such as ethylene oxide, hydrogen peroxide, etc. [54]. Subsequently, cells are embedded into the decellularized ECM, which serves as a scaffolding to support the implanted cells, and this process is known as recellularization [55]. In this review, we will focus on various decellularization procedures and provide a detailed analysis of the most effective approach for decellularizing liver tissue.

## 6. General Decellularization Techniques

**Figure 3:**
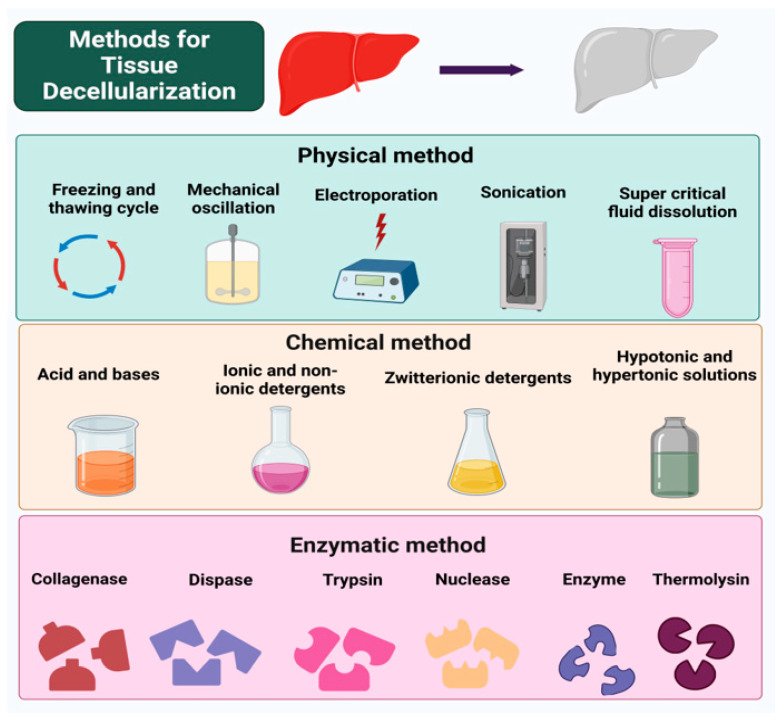
Various decellularization processes for the fabrication of acellular matrices [56,57].

As previously stated, decellularization can be achieved either by chemical, physical, or enzymatic means. Chemical and enzymatic methods are most often used to produce decellularized matrices. The physical approach is used to complement the chemical and enzymatic techniques, as it might have a damaging effect on the matrix. However, chemical techniques may also produce a chemical reaction that might alter the chemical composition of the ECM. Therefore, choosing the right decellularization protocol is necessary based on the application [56,57]. Figure 3 outlines the numerous approaches for developing decellularized extracellular matrix scaffolds.

### 6.1. Chemical Methods

Chemical decellularizing agents can be categorized into surfactants, acids, and bases, of which surfactants are the most commonly employed chemical agents to achieve decellularization [58].

#### 6.1.1. Surfactants

Surfactants can be ionic, non-ionic, or Zwitter-ionic based on their charge [58]. Ionic surfactants work by solubilizing the cytoplasmic components of cells and disrupting the nucleic acids [59]. Sodium dodecyl sulfate (SDS) is the most commonly used ionic agent. They were effective in the complete removal of cellular content. They also facilitated the elimination of about 90% of host DNA. However, they also had detrimental effects on some of the structural and signaling proteins in the ECM [56,58].

Non-ionic surfactants were relatively less strident than ionic decellularizing agents [58]. They cause disruption of the DNA-protein, lipid–lipid, and lipid–protein interactions [59]. The most commonly used non-ionic surfactant is Triton X-100. It was effective in preserving the structural proteins and thereby maintained the integrity and mechanical properties of the tissue [7,58]. However, it was inefficient in completely removing the cellular components from the ECM [59]. It was observed that when employed in conjunction with ammonium hydroxide, DNA elimination occurred to a large extent while the mechanical characteristics of the tissue were also retained [58].

Zwitterionic surfactants exhibit the properties of both ionic and non-ionic surfactants [59]. 3-[(3-cholamidopropyl)-dimethylammonio]-1-propanesulfonate (CHAPS) is generally used. Due to the non-denaturing properties of CHAPS, it is highly efficient in preserving the structural proteins of the ECM, such as collagen and elastin [58,60]. Vacuum-assisted decellularization (VAD) improves the ability of chemical agents to reach the target tissues and thereby enhances the overall efficacy of the procedure [61]. This response could be accounted for by the potential of negative pressures to drive reagents into tissue structures [7,62]. By coupling a vacuum pump to a container housing, the chemical solution, it is possible to create the negative pressures that serve as the driving force of the reagents [62,63]. However, this is not necessarily a decellularization technique but acts as a facilitator and reduces the overall treatment time [64]. Additionally, as indicated by certain research studies, this approach was able to diminish the genetic material within the tissue with no adverse effects on the structural ECM proteins [64,65]. Nevertheless, it is recommended to maintain optimal vacuum duration and pressures because certain studies have indicated disruption of collagen fibrils and increased porosity under prolonged exposure to sub-atmospheric pressures, which corresponds to decreased mechanical properties [33,64,66].

#### 6.1.2. Acids and Bases

Acids cause the dissociation of nuclear DNA from ECM by disrupting nucleic acids and solubilizing cytoplasmic contents [59]. On treatment with peracetic acid, it was observed that the cells were not effectively removed, and the mechanical properties of the tissue, such as stiffness, elastic modulus, and yield stress, were altered. It was observed that the mechanical properties were enhanced, and therefore, this treatment cannot be used for tissues where compliance and expandability are required. Peracetic acid was highly corrosive and oxidizing in nature and, therefore, more often used as a sterilizing agent than as a decellularizing agent [58]. Acetic acid caused the removal of collagen and thereby reduced the overall mechanical strength of ECM; however, it did not affect the sulfated glycosaminoglycans [67].

Treatment with an alkaline solution such as calcium hydroxide was efficient in the removal of cellular and genetic material and also maintained the structural integrity of the tissue. However, the addition of an alkaline solution resulted in swelling of the structure due to the induction of a negative charge on collagen in the tissue, and this swelling caused a reduction in the glycosaminoglycans and, thereby, its viscosity [58]. In addition to the above-mentioned protocols, chelating agents such as ethylene glycol tetraacetic acid (EGTA) and ethylenediaminetetraacetic acid (EDTA) can also be used in decellularization through binding to divalent metal cations and thereby disrupting cell adhesion to ECM [59,68]. Alcohols such as methanol and ethanol can also cause decellularization by dehydration, that is, by replacing the intra-cellular water and therefore disrupting the cell and reducing the content of genetic material in the cell.

### 6.2. Enzymatic Methods

Enzymatic decellularization is often used in conjunction with other methods to assist in the complete removal and degradation of cellular components and nuclear material, respectively, from the ECM. Commonly used enzymatic agents include nucleases, trypsin, collagenase, lipase, dispase, etc. Enzymatic agents such as DNases and RNases function by catalyzing the hydrolysis of deoxyribonucleotide and ribonucleotide chains. Trypsin is the most frequently used proteolytic enzyme to achieve decellularization. It has specific activity on peptides and can have an undesirable effect on ECM components such as collagen, elastin, and GAGs [69]. Therefore, it is usually employed in concert with chelating agents like EDTA and EGTA to prevent undesirable damage to the structural proteins in ECM [58,69]. Collagenase and dispase are scarcely used as they have a direct effect on collagen fibers and may affect the ECM ultrastructure. Furthermore, they do not cause the complete removal of cellular components from the ECM. Therefore, enzymatic agents are generally employed in conjunction with other protocols to achieve effective decellularization [69]. For instance, when SDS, Triton X-100, and peracetic acid/ethanol were used in combination with DNase, it resulted in the effective elimination of cells and genetic material from the ECM while preserving the structural proteins. Thereby, the decellularized matrix displayed similar mechanical properties to that of the native specimen, with its vasculature, ultrastructure, and neural channels intact. The treatment time is of utmost importance and must be carefully monitored since shorter treatment periods may cause ineffective cell removal, while a longer treatment time may result in reduced GAG, collagen, and elastin content [58].

### 6.3. Physical Methods

Physical techniques are not solely employed to achieve decellularization but are used in conjunction with other chemical or enzymatic methods [56]. Some of the techniques that are discussed in this article include freeze–thawing, mechanical loading, hydrostatic pressure, ultrasonication, electroporation, perfusion, and supercritical fluid treatment [61].

#### 6.3.1. Freeze–Thawing

This technique involves cell lysis by alteration of the temperatures between freezing temperatures of about −80 °C and thawing temperatures of about 37 °C. The temperatures to be maintained and the number of cycles to be performed may vary based on the application. For instance, the decellularization of fibroblast cell sheets required three cycles, while lumbar vertebrae cells required only one. The studies reported that collagen and elastin were preserved, but about 88% of the DNA content was also maintained in the fibroblast cells [58]. This proves that freeze–thawing cannot be performed solely as it is not effective in removing the genetic material and, therefore, generally used in assistance with other techniques. A study reported that freeze–thawing, along with using Triton X-100 and sodium dodecyl sulfate detergents on large tendon cells, resulted in about a 20% decrease in DNA content [61]. Thereby, this technique was efficient in preserving the structural proteins; however, it is necessary that it is complemented with certain chemical actions to facilitate effective decellularization.

#### 6.3.2. Mechanical Loading

In this technique, some mechanical stress is applied to the tissues to induce cell lysis. This method typically entails scraping with a sharp instrument to remove the surface cellular components. Given that the underlying components are susceptible to the mechanical force exerted, the amount of force applied should be precise enough [56,61].

#### 6.3.3. Hydrostatic Pressure

This method involves the application of high hydrostatic pressures (HHP) to the tissues to destroy the cell membranes. In some studies, an HHP of about 980 MPa was applied to the porcine cornea for 10 min. It was observed that this treatment had been effective in removing the cell contents; however, it failed to eliminate the nuclear remnants. Therefore, this treatment technique was paired with DNase to completely remove the DNA remnants and thereby prevent immune rejection [58,70]. Several investigations also demonstrated that pressures above 320 MPa increased enthalpy, destabilizing collagen [71]. Very high hydrostatic pressures applied for long durations also pose the problem of denaturing the ECM proteins and altering the mechanical properties of the tissue [58,72]. Therefore, some researchers have performed super cooling pre-treatment before HHP application to weaken the cell membrane and prevent denaturing of tissue structures so that moderately high hydrostatic pressure may suffice to cause effective decellularization [71].

#### 6.3.4. Ultrasonication

High-frequency ultrasonic waves are used to achieve cell isolation by disrupting the inter-molecular bonds and thus lysing the cell membrane. Low-frequency ultrasonic waves can produce some undesirable mechanical effects, such as cavitation, which is associated with bubble formation that may have a damaging effect on the structural and mechanical properties of the tissue [61,73]. Factors such as temperature, presence of dissolved matter, and frequency of ultrasound waves also affect ultrasound cavitation [74]. This treatment technique is collectively used with chemical agents such as SDS and CHAPS to achieve effective decellularization and retain the structural proteins intact. One of the advantages of this procedure is reduced treatment time while achieving high efficiency in the removal of cellular and nuclear material [75].

#### 6.3.5. Electroporation

This technique involves applying microsecond electrical pulses through tissue and destabilizing the cell membrane potential, thereby forming nano-sized pores in the cell membrane and ultimately causing cell apoptosis. Choosing the right electrical parameters is crucial so that no thermal damage is caused to the other structures in the tissue. This decellularization procedure must be carried out in vivo to prevent an immunological response. Due to its non-thermal nature, it is observed that there is no denaturing of the collagen and elastin molecules within the tissue, and the mechanical properties of the matrix remain unaltered [61,76].

#### 6.3.6. Perfusion

Perfusion involves the infusion of chemical agents through the vasculature, which facilitates the removal of cellular and nuclear material. This treatment procedure also preserves the ECM composition and architecture. However, the flow rate must be supervised and controlled as it may damage the capillaries and other vessels [61,72].

#### 6.3.7. Supercritical Fluid Treatment

Supercritical fluid possesses the properties of a normal liquid and a gas; that is, it has a density similar to that of a liquid and a high diffusing capacity similar to a gas [61,77]. In a study involving the decellularization of the optic nerve, it was discovered that the DNA content was reduced by 40% while the GAGs and other structural proteins remained unaltered [77]. Generally, supercritical CO_2_ is used because of its ability to be rapidly removed from the tissue in addition to its non-toxic and non-flammable properties [61,72].

Therefore, it is evident that a variety of procedures may be employed to ensure that the tissue is completely decellularized. A comparison of different methods and the most common applications pertaining to each is illustrated in Table 1 (A–C).

## 7. Suggested Methodology for Optimal Results in Liver Tissue Decellularization

Among the many techniques listed previously, perfusion-based decellularization is reportedly the most popular approach to decellularizing the liver. This technique involves the delivery of chemical and enzymatic agents such as Triton X-100, SDS, EDTA, and others into the portal or hepatic vein in order to efficiently remove cells and create acellular ECM scaffolds [104,105,106,107]. Certain studies have suggested that the process of freezing–thawing may minimize the quantities of decellularization reagents required; nonetheless, cryoprotectants are recommended to prevent any possible damage to the ECM microstructure [5].

Hepatic artery and portal vein cannulations were most frequently performed on rat or porcine livers [108,109,110,111]. A cold NaCl solution and heparin were perfused via the vascular system to remove any remaining blood from the liver [108,110]. The organ was then stored at a temperature of about −80 °C until the decellularization procedure commenced. Prior to starting the procedure, the cryopreserved liver was thawed at a temperature of about 4 °C [108,110,111]. 

**Figure 4:**
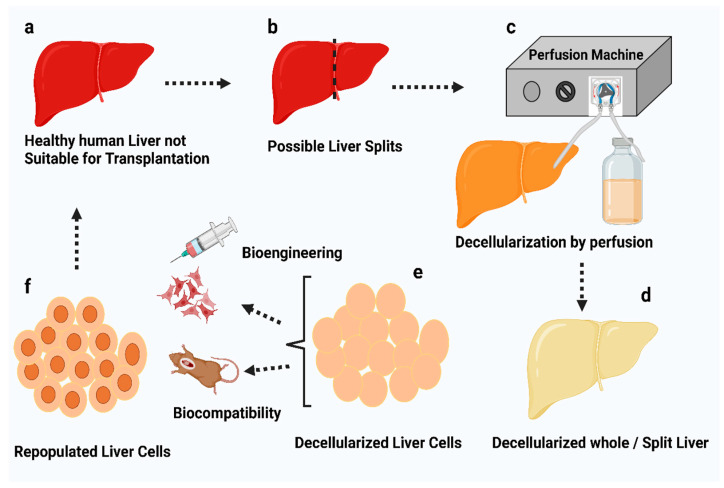
Schematic representation of perfusion-induced liver decellularization. Human livers unfit for transplantation are surgically separated into left lobes or utilized whole (**a**,**b**). Perfusion enables lobes or whole organs to become cannulated and decellularized (**c**) after decellularization the liver cells are dissected by scalpel (**d**).The studies of biocompatibility and bioengineering were analyzed using 3-d techniques (**e**,**f**) [107].

Following this, several studies demonstrated the use of chemical agents such as Triton X-100 or SDS or a combination of both perfused via the hepatic artery or portal vein [6,110,111,112,113]. This was typically carried out iteratively, with the specific chemical detergent being administered initially and continuous perfusion being maintained for around 2 h. The organ was also perfused with distilled water mid-cycle to remove any residual agent and prepare for the next perfusion cycle [110,111]. Finally, the liver was flushed with deionized water and phosphate-buffered saline (PBS) to remove the remnant detergents [110,111,112,113]. A schematic representation of liver decellularization by perfusion is shown in Figure 4.

Perfusion-based decellularization has the advantage of pressure-controlled or flow rate-controlled infusion, allowing for more constant distribution of the chemical agents within the organ [110,111,114]. In a study, it was reported that an average perfusion pressure of 8–12 mm Hg resulted in better preservation of the lobular structures in comparison to the native liver [5,115]. An oscillating pressure system was maintained to simulate the intra-abdominal pressure conditions for more efficient and uniform decellularization throughout the tissue [5,113,116].

## 8. Characterization of Decellularized Liver Samples

**Figure 5:**
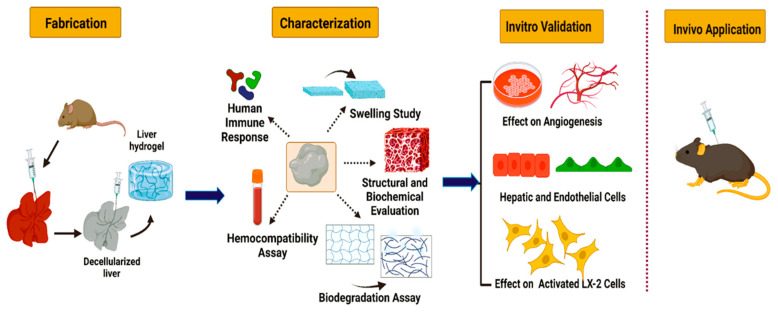
Schematic representation of the decellularization of whole organ liver and the successive characterization techniques [121].

Decellularized liver tissues are preserved in 4% paraformaldehyde to prevent tissue degradation and preserve matrix architecture [110,111,117]. The sample slides were prepared for histology by dehydrating them using graded ethanol, followed by immersion in xylene and embedding in paraffin. In order to evaluate the cellular content and efficiency of decellularization, the samples were stained with hematoxylin, eosin, and 4′,6-diamidino-2-phenylindole (DAPI) [110,111,117]. The DNA content was evaluated with the help of a NanoDrop spectrophotometer based on the measurement of the normalized weight [110,111]. The ECM microstructure was evaluated with the help of scanning/transmission electron microscopy [104,109]. A colorimetric assay was performed to quantify the collagen in the ECM based on the detection of hydroxyproline found in the structural protein [111,118]. Another technique, namely immunohistochemistry, might also be used to indicate the presence of proteins, including collagen type I, collagen type III, collagen type IV, elastin, laminin, and fibronectin [3,5,107]. The GAG content in the ECM was quantified using a glycosaminoglycan assay based on the BlyscanTM dye-binding method [110,119]. The vascular integrity of the decellularized liver scaffold might be assessed by Digital Subtraction Angiography (DSA), with iodine used as the contrast agent delivered via the portal vein [104]. Biodegradation studies were also performed to evaluate the degradation rate of the prepared scaffolds upon incubation in collagenase for a period of about 48 h [120,121]. Figure 5 illustrates the overall fabrication of acellular liver scaffolds and the subsequent characterization procedures to evaluate the decellularization efficiency.

## 9. Effects of Decellularization on ECM

Samples of decellularized liver appeared white and translucent, indicating the removal of cellular material [109,110]. The combinational use of the chemical agents resulted in the removal of a larger percentage of cellular and nuclear material. However, this also reduced the total ECM protein content that is essential for cell adhesion, growth, and mechanical integrity of the matrix [6,110,113]. Some studies revealed an average DNA removal efficiency of greater than 90% for both protocols [110,111]. Certain studies using the Triton X-100 procedure revealed substantially more DNA fragments than the ones using the SDS protocol [122].

Analytical procedures revealed that ECM proteins such as collagen and sGAGs were present in larger concentrations than in native liver tissue due to the elimination of cellular debris [113,120]. However, it was shown that the elastin content in the acellular liver was slightly lower than in fresh liver samples [107,120]. When examined using a scanning/transmission electron microscope, the 3D architecture and ultrastructure of the ECM were discovered to be intact, suggesting that the connective fibers retained the polygonal-like architecture and the ECM structural proteins, including collagen, fibronectin, and laminin were also conserved [104,107,109,116,123]. According to Hussein et al., 77% of collagen degradation occurred during the first three hours of placing the samples in collagenase. However, Baptista et al. reported 80% collagen degradation within the first six hours of placement and complete degradation in 48 h, indicative of the possible instability of the acellular samples upon enzymatic action [120,121].

Following decellularization using the suggested procedure, an intact vasculature was also observed, which is essential to maintain the delivery of growth factors, nutrients, and oxygen to the newly repopulated cells. [104,116,120]. However, certain studies have indicated possible ECM damage at higher SDS concentrations; therefore, lower proportions for longer durations were recommended [113]. Table 2 presents a comparative analysis of different techniques conducted under varying conditions and their impact on ECM.

## 10. Applications of Various Decellularized Liver Matrices and the Techniques Involved

Liver decellularized matrix is a promising biomaterial for fabricating 3D-bio printed, nanoparticle-incorporated, electrospun, and freeze–dried scaffolds. This is attributed to the microenvironment created by the matrix that closely resembles that of the native tissue, thereby promoting cellular functions such as proliferation and differentiation [131,132].

### 10.1. 3D Bioprinting of Decellularized Hepatic Extracellular Matrix

**Figure 6:**
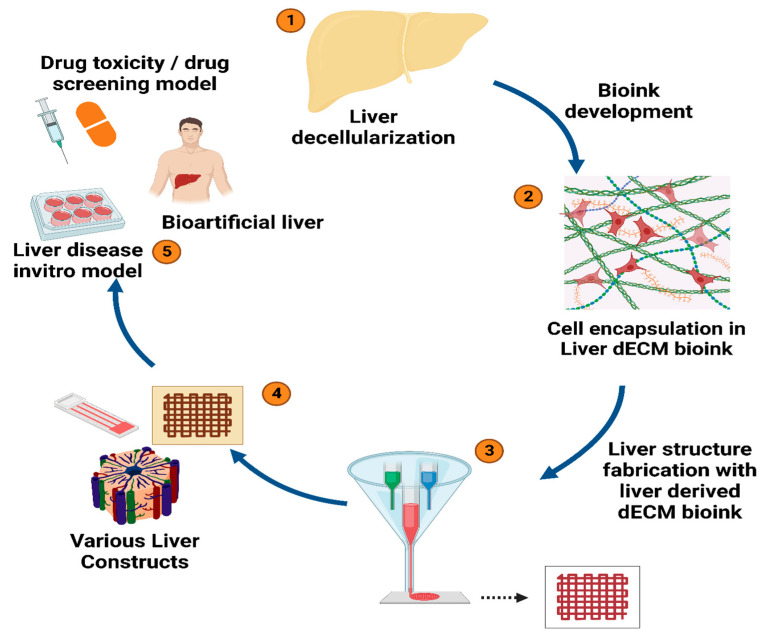
Illustrates the bioprinting approach using decellularized liver ECM bioink to create 3D liver constructs [135].

The use of 3D bioprinting involves layer-by-layer deposition of a material, permitting the fabrication of a highly controlled and desired 3D structure with improved resolution [132,133,134]. This method is extensively used because of its ability to mimic the complex, intricate structure of the liver ECM and its wide usage with a variety of biomaterials and cell types [135,136]. Additionally, the 3D structures offer biomechanical and biochemical cues stimulating numerous cellular processes since they preserve the native ECM and its structural integrity [137,138]. In this procedure, a bioink is developed, which is essentially a compound of suitable biomaterial and decellularized liver matrices (Figure 6) [134,135]. These materials are reinforced into the decellularized liver ECM for enhanced support, mechanical stability, printing resolution, rheological properties, and bioink ejection [139,140,141].

It is important to choose appropriate biomaterials that demonstrate biocompatibility, printability, sufficient mechanical strength, and resemblance to native liver morphology [139,141,142]. Naturally derived biomaterials such as collagen, gelatin, agarose, chitosan, hyaluronic acid, and others are most commonly used to prepare bioinks [135,142]. To prepare the bioink, the decellularized liver matrix is first lyophilized and powdered, then dissolved in acidic solutions of appropriate concentrations, such as 0.1 M acetic acid or 0.1 N hydrochloric acid, for around 4 days. Subsequently, the obtained solution is centrifuged at 3000–3500× *g* rpm for around 10 min to eliminate larger particles. The pH of the resultant solution can be modified to 7.4 by adding 10 M sodium hydroxide (NaOH) or phosphate-buffered saline (PBS) solution [135,138,140]. Similarly, bio-inks made of gelatin, collagen, and other materials, or their combinations, can be made by dissolving the corresponding components in appropriate solutions (for instance, collagen in 0.5 M acetic acid) and correcting the pH of the resulting solution with 10 M NaOH solution. Finally, the desired bioink may be prepared by combining the acquired mixture with the decellularized liver ECM bioink [135,143,144,145].

The prepared bioink loaded into a syringe is extruded out of the nozzle using pneumatic or mechanical forces and deposited over the print bed to create 3D structures [133,134]. Certain studies have utilized UV light for crosslinking to facilitate a more uniform and better printing resolution; however, this resulted in an alteration in cell behaviors upon extended usage [135,138,139].

### 10.2. Nanoparticles-Incorporated Decellularized Extracellular Matrices

Nanoparticles may be integrated into decellularized liver scaffolds for enhanced healing of damaged tissue, cellular growth, structural stability, antibacterial activities, and sustained release of growth factors [146,147,148]. Additionally, certain nanomaterials, such as polydiacetylene (PDA), may be reinforced into decellularized liver matrices to facilitate blood detoxification due to their antidotal behavior [149]. These nanoparticles are claimed to bind to matrices by ionic or covalent bonding, where the latter is dependent on the functional groups present on their surface [148].

**Figure 7:**
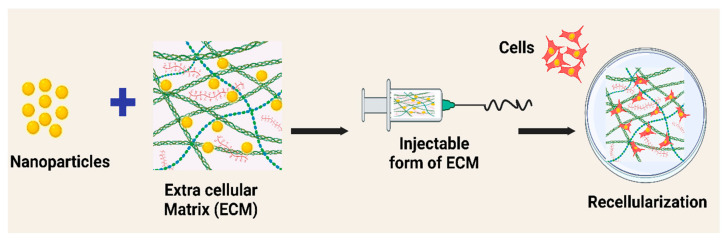
Schematic illustration of the extracellular cellular matrix-nanoparticles conjugated system and its numerous advantages, including improving structural stability and electrical conductivity, sustained release of growth factors, improving cellularity, adding antibacterial activities, and antitoxin functionalization [147].

Various studies have tested the functioning of silver nanoparticles conjugated in the matrices, and it is reported that they facilitated crosslinking due to their high affinity for collagen, thereby increasing the overall structural stability and resistance to biodegradation in vivo. Moreover, such conjugated scaffolds have demonstrated biocompatibility, anti-inflammatory activities, cytocompatibility, and less immunogenicity [147,148]. Figure 7 depicts the nanoparticles-incorporated extracellular matrix system and its overall benefits.

### 10.3. Electrospinning

**Figure 8:**
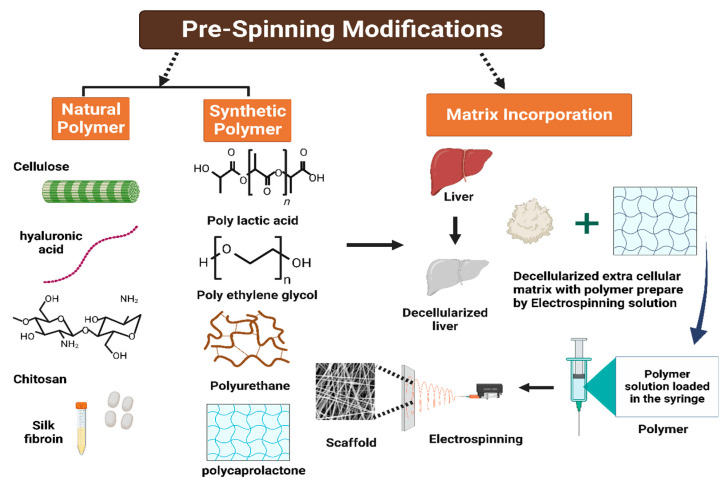
General scheme of scaffold fabrication using electrospinning and decellularized extracellular matrix–polymer blend solution [155].

Electrospinning is a prevalent technique to produce micro- or nanofibers with precisely controlled diameters and a high surface-to-volume ratio, mimicking the structure of the extracellular matrix (ECM) in native tissues [150,151,152]. The fabrication of electrospun fibrous scaffolds using decellularized extracellular matrices blended with polymers reportedly produced design-driven constructs that are capable of retaining tissue-specific phenotypes, enhancing the proliferation and differentiation of seeded cells [153]. Figure 8 shows the general scheme of scaffold fabrication using decellularized ECM and electrospinning. 

Decellularized porcine liver extracellular matrix (PLECM) and collagen type I were dissolved in 1,1,1,3,3,3-hexafluoro-2-propanol (HFIP) solvent and electrospun into porous 3D nanofibrous scaffolds. These scaffolds were then used to culture Primary Human Hepatocytes (PHH) alone or in combination with non-parenchymal cells such as 3T3-J2 murine embryonic fibroblasts and liver sinusoidal endothelial cells. Cells cultured on the fibrous scaffolds displayed superior urea synthesis, albumin secretion, and cytochrome-P450 1A2, 2A6, 2C9, and 3A4 enzyme activities compared with those cultured on conventionally adsorbed 2D ECM controls [154].

Another study incorporated a decellularized human liver ECM directly into the fibers of an electrospun polylactic acid (PLA) matrix to create a bioactive protein–polymer scaffold to enhance the proliferation of THLE-3 hepatocytes [156]. A different method involved seeding a layer of bladder epithelium that produces ECM onto electrospun polycaprolactone (PCL) fibers to create hybrid polymer–ECM scaffolds. The initial layer was removed through detergent-based decellularization, and liver-representative cells (HepG2) were then grown on the scaffolds. The use of such scaffolds has proven to have a positive impact on the gene expression profile, albumin production, attachment, and survival of liver cells, which cannot be achieved by individual ECM components alone [153,157].

### 10.4. Lyophilization

Lyophilization or freeze–drying, a well-known method for fabricating scaffolds with controllable porosities, has been employed to enhance the shelf-life of decellularized heart valves in sheep [158]. A renal matrix scaffold was fabricated by extracting sheep kidney cells through decellularization followed by freeze-drying and chemical crosslinking in order to enhance the mechanical properties and pore structure [37,159,160].

**Figure 9:**
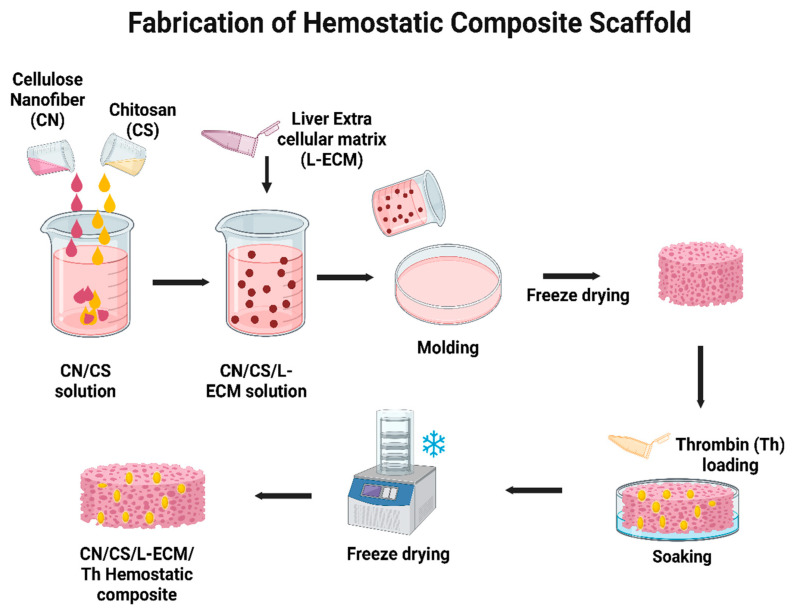
Schematic representation of the fabrication of CN/CS/ECM-Th Hemostatic composite using freeze drying [161].

In another study, researchers explored the possibility of developing a hemostatic and liver wound-healing nanocomposite material using a non-solubilized, powdered, decellularized liver extracellular matrix (L-ECM). The L-ECM was anchored to thrombin by lyophilization, and a TEMPO-oxidized cellulose nanofiber/chitosan/ECM-thrombin nanocomposite was developed. Figure 9 represents the procedure for the fabrication of the CN/CS/ECM-Th scaffold by means of the freeze–drying approach. The resultant biomaterial exhibited rapid pro-coagulation ability, highlighting its potential as a liver regeneration scaffold [161].

## 11. Conclusions

The process of tissue decellularization, which belongs to the broad categories of tissue engineering and regenerative medicine, has garnered significant attention due to its ability to generate scaffolds that closely mimic the biological environment of the human body. This advancement retains the potential for enhancing the efficacy of tissue regeneration, restoration, and replacement. In recent years, the scarcity of available donor organs and the potential risks of immunogenic rejections post-transplantation have prompted researchers to explore alternative approaches for tissue repair and regeneration. One such approach acquiring significant attention is the utilization of decellularized tissues, which harness the patient’s own cells for these purposes. This article aims to delve into the growing interest in decellularized tissues as a potential solution to address the aforementioned challenges in the field of transplantation and tissue engineering. The efficacy of tissue regeneration is dependent upon the successful elimination of cells and the triggering substances that induce an immunological response. In spite of the diverse range of methods available for cell isolation and scaffold preparation, the selection of an appropriate decellularization technique holds significant importance. The selection of an appropriate decellularization procedure is crucial and should be tailored to the specific tissue or organ under investigation, taking into account its unique extracellular matrix (ECM) structure and composition. The present study aims to evaluate the effectiveness of perfusion as a widely utilized process for achieving successful decellularization of the liver while preserving the organ’s structural integrity. The present study aims to investigate the efficacy of a particular procedure in eliminating cellular and nuclear material. Multiple reports have indicated that this procedure exhibits promising results in achieving this objective. However, it is evident from various reports that although this procedure has been successful in eliminating the cellular and nuclear material to a large extent, care must be taken to ensure the chemical agents are introduced at appropriate concentrations to prevent any damage to the ECM microarchitecture. Therefore, it is imperative to conduct further investigations in order to substantiate the current knowledge and expedite the development of promising decellularization techniques, enabling preferable alternatives to conventional medical therapies.

## Figures and Tables

**Table 1:** (A) A comparative study of the various decellularization procedures using physical methods and their applicability to various tissues. (B) A comparative study of the various decellularization procedures using enzymatic methods and their applicability to various tissues. (C) A comparative study of the various decellularization procedures using chemical methods and their applicability to distinct tissues.

Technique	Mechanism of Action	Effects	Advantages	Limitations	Applications	References
(A)						
Freeze–thawing	denatured proteins, resulting in cell necrosis.	eliminated cellular contents completely. retained structural proteins.	adequate cell removal. intact basement membrane.	inefficient removal of genetic material. Ice crystal formation may disrupt the ECM ultrastructure.	tendon, porcine carotid artery, porcine renal tissue.	[78,79,80,81,82]
Mechanical Loading	application of physical force caused cell lysis.	removed the surface cellular components of the tissue or the whole organ.	minimal disruption to the ECM architecture.	only suitable for tissues with sufficient mechanical hardness and a less dense ECM. applying force must be performed with the utmost caution to prevent damage to interior structures.	small intestine, urinary bladder	[56,61]
Hydrostatic pressure	high hydrostatic pressure caused cell membrane disruption resulting in cell death.	effectively removed cellular and nuclear content.	biomechanical properties of decellularized grafts remain unaltered. relatively short treatment time.	ultra-high pressures can result in protein denaturation.	blood vessels, uterine tissue, and corneal tissue.	[70,71,83]
Ultrasonication	caused cell membrane disruption due to induced shear stresses by cavitation.	effective removal of about 90% of cellular content. preserved structural proteins of cells.	retained the ECM microstructure. short treatment time.	demand perfect control over sonication power.	umbilical artery, aorta	[75,84,85,86]
Electroporation	distortion of the cell membrane occurred following the application of pulsed electric fields.	efficient cell removal. ECM remained intact.	porosity can be controlled by adjusting the electrical parameters. intact basement membrane.	relatively smaller electrodes limit the tissue surface area decellularized. not preferable for cardiac applications.	porcine liver, skin tissue.	[61,76,87,88]
Perfusion	eliminated the cell remnants by allowing a constant flow of decellularizing agents through the tissue.	solubilized cellular and nuclear material. Preserved structural proteins.	generation of biocompatible, non-toxic decellularized scaffolds. maintained ECM ultrastructure.	size shrinkage occurred following the procedure.	porcine renal tissue, heart, and lung tissue.	[89,90,91,92]
Supercritical fluid	A relatively inert gas, carbon dioxide at low temperature and pressure conditions facilitated the removal of cellular components.	effectively removed cellular and immunogenic agents. cause tissue dehydration.	preserved the structural integrity of ECM proteins. easily achievable treatment conditions with relatively brief treatment times.	tissue dehydration results in increased scaffold brittleness.	bovine neural tissue, porcine cartilage, adipose tissue, and bovine dermis.	[77,93,94,95,96]
(B)						
Nucleases (DNases and RNases)	disintegrated nucleic acid sequences by cleaving the phosphodiester bonds.	eliminated cellular remnants. retained collagen and elastin	retained the biomechanical properties of the original tissue	ineffective in the complete removal of nuclear material. Induced immunological response	neural tissue, trachea, adipose tissue, intervertebral discs, porcine heart valves	[7,58,59,67,97,98]
Proteases (Trypsin, collagenase and dispase)	cleaved peptide bonds selectively.	detached cells from the tissue. removed matrix proteins such as collagen, elastin, laminins, etc.	efficient in complete cell removal in soft tissues. impedes cell conglomeration.	disrupted the ECM integrity. reduced biomechanical strength of decellularized scaffolds. Ineffective in complete cell removal.	porcine cornea, heart valves, and the dermis.	[7,67,68,69,97,99,100]
(C)						
**Surfactants**						
Ionic (SDS)	solubilized the cell and nucleic materials Denatured proteins	effectively removed cellular and nuclear material. distorted the structural and signaling proteins.	allowed for complete cell removal and about 90% of host DNA.	disrupted the ECM. decreased GAGs and growth factors. cytotoxic and required an extensive wash process.	porcine cornea, porcine myocardium, porcine kidney, human vein, etc.	[58,59]
Non-ionic (Triton X-100)	disrupted DNA-protein, lipid-protein, and lipid-lipid interactions	partially efficient in removing genetic material. Retained elastin.	less harsh than SDS and, therefore, caused less damage to the structural integrity of ECM.	less effective than SDS in removing cells and nuclear material. should be used in conjunction with Ammonium Hydroxide to facilitate complete cell removal.	bovine pericardium, porcine kidney, etc.	[58,59,101]
Zwitterionic (CHAPS)	solubilized cell and nuclear membranes Exhibited properties of both ionic and non-ionic surfactants	preserved structural proteins. Effectively removed about 95% of nuclear constituents.	superior cell removal. substantial preservation of ECM architecture.	disrupted the integrity of the basement membrane of ECM	vasculature, neural tissue.	[59,60,102]
Acids and Bases	hydrolyzed cytoplasmic constituents of cell	reduced collagen, GAG content, and growth factors.	treatment with an alkaline solution allowed for the complete removal of cellular and nuclear material. acid-mediated decellularization was effective in eliminating residuary genetic constituents.	effects on ECM were found to be strident disrupting the peptide bonds and reducing the overall mechanical properties.	small intestine submucosa, urinary bladder matrix, and dermis samples.	[53,56,58,67,102,103]

**Table 2:** A comparison of various protocols implemented under various settings and their implications on liver ECM.

Protocol	Effects on ECM	References
1% Triton X-100	adequate clearance of cellular debris, a high DNA removal efficiency of about 96%, better collagen retention	[110,124]
0.1% SDS	high cell elimination efficacy complete removal of genetic material retained the structural proteins and integrity of the ECM	[105,124,125]
1% SDS	complete cell removal highly efficient in DNA removal with an efficiency of about 99% disrupted the microvasculature of ECM	[111,113,122]
1% Triton X-100 + 1% SDS	effective removal of cellular components and complete elimination of nuclear material preserved the vasculature and mechanical integrity of the ECM	[113,126]
4% Triton X-100/0.02% EGTA solution and 0.5% SDS aqueous solution	Decellularized whole liver organ as an ex vivo model with a unique native environment and vasculature for vascular embolization evaluation.	[127]
Free-thaw + Triton X-100/SDS + DNase/RNase	Sequentially perfusing the organ with SDS and Triton X-100, resulting in the generation of translucent acellular liver matrices within just 9 h. This approach offers a more streamlined and effective method for decellularization.	[128]
1% Triton X-100 + 0.05% EDTA + 30 μg/mL DNase	A unidirectional, one-way perfusion flow improved and accelerated the decellularization approach. Most significantly, decellularization preserved liver extracellular matrix integrity and cell adhesion and proliferation, enabling recellularization.	[129]
Enzymatic	Utilizes enzymes (e.g., trypsin, DNase) to digest cellular material while leaving the ECM intact.	[120]
Physical	Involves physical disruption of cells through mechanical agitation, shear, or pressure to remove cellular material.	[130]

## Data Availability

Data sharing is not applicable since no new data were generated.
